# *In Vitro* and *In Vivo* Comparison of Different Types of Rabbit Mesenchymal Stem Cells for Cartilage Repair

**DOI:** 10.22074/cellj.2019.6149.

**Published:** 2019-02-25

**Authors:** Mohammad Ali Khalilifar, Mohamadreza Baghaban Eslaminejad, Mohammad Ghasemzadeh, Samaneh Hosseini, Hossein Baharvand

**Affiliations:** 1 Department of Stem Cells and Developmental Biology, Cell Science Research Center, Royan Institute for Stem Cell Biology and Technology, ACECR, Tehran, Iran; 2 Department of Developmental Biology, University of Science and Culture, Tehran, Iran; 3 Infertility and Reproductive Health Research Center, Health Research Institute, Babol University of Medical Sciences, Babol, Iran

**Keywords:** Articular Cartilage, Mesenchymal Stem Cells, Rabbit, Transplantation

## Abstract

**Objective:**

Systematic studies indicate a growing number of clinical studies that use mesenchymal stem cells (MSCs) for the treatment of cartilage lesions. The current experimental and preclinical study aims to comparatively evaluate the potential of MSCs from a variety of tissues for the treatment of cartilage defect in rabbit’s knee which has not previously been reported.

**Materials and Methods:**

In this experimental study, MSCs isolated from bone marrow (BMMSCs), adipose (AMSCs), and ears (EMSCs) of rabbits and expanded under *in vitro* culture. The growth rate and differentiation ability of MSCs into chondrocyte and the formation of cartilage pellet were investigated by drawing the growth curve and real-time polymerase chain reaction (RT-PCR), respectively. Then, the critical cartilage defect was created on the articular cartilage (AC) of the rabbit distal femur, and MSCs in collagen carrier were transplanted. The studied groups were as the control (only defect), sham (defect with scaffold), BMMSCs in the scaffold, EMSCs in the scaffold, and EMSCs in the scaffold with cartilage pellets. Histological and the gene expression analysis were performed following the transplantation.

**Results:**

Based on our comparative *in vitro* investigation, AMSCs possessed the highest growth rate, as well as the lowest chondrogenic differentiation potential. In this context, MSCs of the ear showed a significantly higher growth rate and cartilage differentiation potential than those of bone marrow tissue (P<0.05). According to our *in vivo* assessments, BMMSC- and EMSC-seeded scaffolds efficiently improved the cartilage defect 4 weeks post-transplantation, while no improvement was observed in the group contained the cartilage pellets.

**Conclusion:**

It seems that the ear contains MSCs that promote cartilage regeneration as much as the conventional MSCs from the bone marrow. Considering a high proliferation rate and easy harvesting of MSCs of the ear, this finding could be of value for the regenerative medicine.

## Introduction

The treatment of articular cartilage (AC) injuries is one of the major challenges in orthopedics. Despite encouraging results of the current approaches in the elimination of symptoms of cartilage lesions, the newly- formed tissue is not similar to normal hyaline cartilage in terms of biomechanical properties and the longterm durability. Therefore, it is necessary to develop a biological solution to achieve maximum quality of new AC with the long-term effect (1). 

AC is structurally composed of four areas: the superficial area, middle area, deep area, and calcified area (1). The extracellular matrix feature, chondrocyte phenotype, and the cell shape vary among the different areas (2). AC is referred to the as hyaline cartilage that covers the end of bones and forms diarthrodial joints acting as a shock reducer and a lubricant. AC is a kind of tissue where the cellular matrix shows a collapsed structure lacking lymphatic, blood, and nerve supply, and contains a minimum number of chondrocytes (3). Thus, it has limited the intrinsic regeneration capacity (4). Accordingly, the untreated defects lead to osteoarthritis (OA) and joint degeneration. OA causes the disruption of the collagen networks and proteoglycan depletion of AC. In addition to AC, OA involves in the other joint tissues such as the synovium, meniscus, and subchondral bone (5). Therefore, successful treatment of cartilage defect is essential to prevent the progression of cartilage destruction. 

There are different strategies for cartilage defect treatment, yet each procedure possesses several limitations. Debridement and lavage are appropriate for the chondral lesions smaller than 2 cm in diameter. Microfracture is used for the cases with small chondral lesions (smaller than 2-3 cm in diameter), but the newly-formed tissue is fibrocartilage (6). Donor limitation and donor site morbidity are the limitations with respect to the use of autografts or mosaicplasty. This technique could cover maximum 3-4 cm of a defect. Osteochondral allograft transplantation is commonly used for the extended osteochondral defects; however, the tissue adaptability and limited availability are the most restrictions of this method. In 1987, Brittberg introduced the autologous chondrocyte implantation (ACI) for the treatment of full-thickness defect (7). Recent studies have reported the advantages of ACI versus microfracture, but, in spite of using third-generation of ACI, it has own drawbacks. The requirement for a two-stage surgery, expansion under *in vitro* culture, dedifferentiation after implantation and inability to treat large chondral defects due to donor site deficit and morbidity are some of the drawbacks for the use of chondrocytes related to ACI (8). To overcome the limitations of current approaches, tissue engineering with three basic parts, cells, scaffolds, and biological signaling molecules have emerged as an alternative strategy to repair cartilage efficiently (9). Furthermore, multiple studies have so far been conducted to improve the AC injuries, using a variety of cells worldwide (4).

A proper cell source should meet several criteria such as easy accessibility, expansion, differentiation capacity, and the lack of tumorigenic and immunogenic properties. Embryonic stem cells (ESCs), induced pluripotent stem cells (iPSCs), committed chondrocytes, and adult stem cells are the candidate cell sources for clinical application. ESCs and iPSCs are associated with the ethical and tumor formation concern. Chondrocytes have limited redifferentiation capability, while the adult stem cells which can be obtained from different adult tissues would be a promising cell source (10). The ease of separation and expansion, multipotency and capability to differentiate into mesodermal and nonmesodermal lineages, low immunogenicity, and secretion of trophic factors by MSCs have attracted great attention for the future cell- based approaches (11-14). Studies of cartilage repair using MSCs have mainly focused on the application of bone marrow mesenchymal stem cells (BMMSCs). It has been shown that differentiation into chondrocyte is induced by some growth factors (15-17). Numerous clinical studies have demonstrated the positive effect of BMMSCs in AC regeneration (18). In recent years, MSCs isolated from adipose tissue (AMSCs) have been considered a potent alternative due to their availability and minimal donor tissue morbidity (9). AMSCs have been applied to regenerate cartilage defects (19), and comparison between BMMSC and AMSC in differentiation potential to chondrocyte was also investigated (9, 20). Moreover, ear-derived MSCs (EMSCs) showed the differentiation capability into osteocytes, chondrocytes, and adipocytes (21). 

Seeding of MSCs onto diverse scaffolds such as collagen is an effective method used to deliver MSCs into cartilage defects. The ideal scaffold, in addition to keeping implanted MSCs inside cartilage lesions, should provide the bioactive compounds necessary for the induction of differentiation and maturation of MSCs (22). In this study, for the first time, an attempt was made to compare the differentiation ability, and regenerative potential of MSCs derived from bone marrow, adipose, and the ear to chondrocytes *in vitro*. Furthermore, we evaluated the regenerative potential of a construct comprised of commercially-available collagen type I (as a scaffold) loaded with MSCs from bone marrow and the ear (as a cellular component), and cartilage pellets (as a biological signal) in rabbit’s AC defects.

## Materials and Methods

### Rabbits

In this experimental study, skeletally matured New Zealand white rabbits (*Oryctolagus cuniculus*) were provided by the animal house of Royan Institute, Tehran, Iran. The Rabbits were used in the experiments weighing approximately 2.7 kg (ranging from 2.1 to 3.1 Kg). The animal care was done in accordance with the animal house guidelines and approval from the Ethics Committee of the Royan Institute. Eighteen white rabbits were generally anesthetized by one dose of an intramuscular injection of 35 mg/kg ketamine and 10 mg/kg xylazine mix (ketamine HCL 100 mg/ml and xylazine HCL 20 mg/ml, Alfasan, Holland). The animals were kept in one cage while they were free to move. 

### Isolation and culture of rabbit’s ear mesenchymal stem cells

A small piece of the ear (1 cm diameter) without large blood vessels was punched under anesthesia. The wound area was disinfected with oxytetracycline spray after punching, and because of the high intrinsic regeneration potential of rabbit ear, the healing occurred after 8 weeks (Fig .S1) (See Supplementary Online Information at www.celljournal.org). The outer layers of the skin and connective tissues were removed, and the remaining cartilage was washed with PBS, and then, chopped. Cartilage was digested with 5 mg/ml collagenase type I (Sigma-Aldrich, USA) in phosphate buffered saline (PBS) at 37°C for three hours. The isolated cells were cultured in Dulbecco’s Modified Eagle Medium (DMEM, +4500 mg/L Glucose, Gibco, USA) supplemented with 10% fetal bovine serum (FBS, Gibco, USA) and Pen/Strep (50 U/ml penicillin+50 µg/ml streptomycin, Pen/Strep (Gibco, USA) and incubated at 37°C with humidified 5% CO_2_. The medium was changed after 48 hours to remove non-adherent cells. Adherent cells were cultured till reached 80% confluent (23). The cells were removed by trypsin-EDTA (0.05% trypsin-EDTA, Gibco, USA) and passaged to a 75-cm^2^ flask (TPP, Switzerland). The cells proliferated until passage three. 

### Isolation and culture of bone-marrow mesenchymal stem cells of rabbits

The knees of anesthetized rabbits were shaved and disinfected with Savlon surgical scrub. Bone marrow was harvested under aseptic conditions from the tibia. Specimens were cultured in a 25 cm^2^ culture flask that contained 4 ml DMEM low glucose with 10% FBS and Pen/Strep (50 unit/ml penicillin+50 µg/ml streptomycin). The flask was incubated at 37°C with humidified 5% CO_2_. After 2 days, the medium was changed to remove non- adherent cells, and the adherent cells were cultured till reached 80% confluent. The culture medium was changed every two days, and the cells proliferated until passage three.

### Isolation and culture of adipose mesenchymal stem cells of rabbits

We obtained the adipose tissue from the fat pad located subcutaneously between the scapulae of rabbit and chopped well as previously described (24). The tissue was digested in 5 mg/ml collagenase for three hours at 37°C under constant agitation. The digested tissue passed through 70-micron nylon filter mesh followed by centrifugation at 1500 rpm. The cell pellet was cultured in DMEM culture medium. After 48 hours, the medium was changed to discard non-adherent cells; the adherent cells were cultured for the next seven days by changing the medium twice weekly. The cells proliferated until passage three. 

### Tri-lineage cell differentiation

To prove the mesenchymal phenotype of the isolated cells, passage-3 cells were differentiated into adipogenic, chondrogenic, and osteogenic lineages. 0.3×106 cells were seeded per well of a 6-well culture plate. For osteogenic differentiation, the medium was replaced by osteogenic medium-DMEM supplemented with 50 mg/ml ascorbic acid 2- phosphate (Sigma, USA), 10 mM ß glycerol phosphate (Sigma, USA) and 10 nM dexamethasone (Sigma, USA). After two weeks, the medium was discarded and cell monolayers were fixed with methanol, and then, stained with alizarin red.

To induce adipogenesis, the adipogenic medium that contained 100nM dexamethasone (Sigma, USA) and 50 mg/ml indomethacin (Sigma, USA), 100 µM L-Ascorbic acid (Sigma, USA) was added to each well. At day 21, the culture medium was removed and the cells were fixed with 4% formalin at room temperature for 1 hour, and then, stained with oil red solution in isopropanol 99% for 15 minutes. The light microscope was used to visualize the adipose droplets.

A micro-mass culture system was used to induce chondrogenic differentiation of MSCs. Briefly, 2.5×105 passaged-3 cells were pelleted under 400 g for 10 minutes and cultured in chondrogenic medium (high glucose DMEM supplemented by 10 ng/ml transforming growth factor-ß3 (TGF- ß3, Sigma, Germany), 10 ng/ml bone morphogenetic protein-6 (BMP6, Sigma, Germany), 1:100 diluted insulin transferrin selenium+premix (Sigma, Germany, 6.25 µg/ml insulin, 6.25 µg/ml transferrin, 6.25 ng/ml selenious acid, 1.25 mg/ml bovine serum albumin, and 5.35 mg/ml linoleic acid, and 10% FBS) for 21 days at 37°C, 5% CO_2_; with medium change twice weekly. Chondrogenic differentiation was assessed by both toluidine blue and Verhoeff-van Gieson staining of pellet sections. The sections were hydrated and stained, using toluidine blue for 30 seconds at room temperature for showing proteoglycan subunits in the extracellular matrix which is one of the characteristics of hyaline cartilage. The pellet sections were also stained with Verhoeff-van Gieson that is useful in demonstrating of elastic fibers which are abundant in elastic cartilage such as the ear and invisible in hyaline cartilage. 

For the cartilage production as previously reported (25), third passaged cells were suspended in the chondrogenic medium at 2×10^7^ cells/ml. Droplets (12.5 µl) were carefully placed at the bottom of each well of a 96-well plate. Cells were allowed to adhere at 37°C for 2 hours, followed by the addition of 200 µl chondrogenic medium incubated at 37°C with 5% CO_2_ and 80% humidity. After 24 hours, the cells of the droplets joined together and became spherical. The medium was changed every 3 days, and micro masses were harvested on days 21, for transplantation in defects.

### Growth rate and proliferation

To study the growth rate and proliferation velocity of MSCs from three different tissues, the growth curves for these cells were plotted. 104 passaged-3 cells of each group were seeded per well of a 24-well plate. Every day, the cells from two wells were harvested and singled with trypsin/EDTA, treated with diluted trypan blue and counted unstained cells with Neubauer slide under a light microscope until day 12. The culture medium was DMEM with 10% FBS and 100 U/ml pen/strep that was changed every two days. We calculated the population doubling time (PDT) using the following formula: DT=T ln2/ln (Xe/Xb). 

### Quantitative real-time polymerase chain reaction analysis 

The expression levels of chondrogenic [*SOX9*, *COL2a1*, and AGGRICAN (*ACAN*)], adipogenic [lipoprotein lipase (*LPL*), adiponectin (*ADIPOQ*) and PPARG], and osteogenic markers (*OCN, OPN, ALP*, and *COL1a1*) were evaluated using quantitative polymerase chain reaction (qPCR) (23). The list and sequences of primer pairs are provided in Appendix Table S1 (See Supplementary Online Information at www.celljournal. org). Trizol reagent was used for total RNA extraction according to the manufacturer’s instructions (Sigma, USA). cDNA was synthesized (Eppendorf mastercycler gradient, Germany) according to the cDNA Reverse Transcription Kit protocol (Sina Clone, Iran). The PCR was performed with SYBR Green universal PCR Master Mix (Applied Biosystems StepOnePlus TM Real- time PCR System, USA) with a real-time PCR system (Applied Biosystems Life Technologies, Inc., ABi StepOnePlus) and analyzed with Step One software (Applied Biosystems, version 2.1).

Glyceraldehyde-3-phosphate dehydrogenase (*GAPDH*) primers were utilized as an internal control. To calculate the fold change, the ..CT method was used, and all values were normalized against undifferentiated MSCs.

### Animal studies

The rabbits were generally anesthetized and legs prepared as described previously (26). Briefly, a 4cm medial parapatellar incision was made over the knees, and the patella retracted. We used a hand drill (trephine drill, 369.05, A. TITAN, USA) to create a critical defect (4.5 mm in diameter and depth of 1 mm) on the AC of the patellar groove of the distal femur (27) (Fig .S2) (See Supplementary Online Information at www.celljournal.org). Collagen type 1 scaffold that was used in this study was commercially-available and purchased from Koken Cellgen, Collagen solutions for tissue culture, Japan. The rabbits were divided into different groups including the negative control (defect without any treatment), sham group (defects filled with only collagen type 1 scaffold), the group that was transplanted with 10^6^ BMMSCs in scaffold, the group that was received 10^6^ EMSCs in scaffold, and the group that was implanted with 10^6^ EMSCs in scaffold along with several cartilage pellets. The animals were anesthetized 4 and 8 weeks post-surgery with an intramuscular injection of 35 mg/kg ketamine and 3 mg/kg xylazine and then euthanized with saturated KCl heart injection. Rabbit knees were removed and prepared for macroscopic and microscopic evaluations. 

To detect MSCs in recovered defects, BMMSCs were labeled with PKH26 red fluorescent cell membrane linker, a vital dye for *in vivo* cell tracking studies (MINI26, Sigma-Aldrich, Germany)

### Macroscopic and microscopic evaluations 

Macroscopic evaluation: the removed knees were numbered in a histological laboratory on a clean cloth and photographed. The filling rate, color, and surface mode of the repaired defect of the knees were scored blindly according to the scoring system identified by Rudert et al. (28) (Table S2) (See Supplementary Online Information at www.celljournal.org). 

Microscopic evaluation: to histologically evaluate the degree of regeneration in damaged cartilage, all femoral condyles were trimmed and fixed in 10% buffered formalin for 48 hours. The tissues were decalcified using 5% formic acid in distilled water for 7 days. The decalcified tissue was dehydrated with 60-100% ethanol, immersed in xylene, and finally embedded in paraffin. At two different levels, from anterior to posterior, 5 µm thick paraffin sections were cut from transverse femoral condyle and stained with toluidine blue and hematoxylin-eosin (H&E). These sections were scored by two pathologists using the criteria reported by Wakitani et al. (29) containing matrix-staining, surface regularity, cell morphology, the thickness of cartilage (%), and integration with adjacent cartilage (Table S3) (See Supplementary Online Information at www.celljournal.org).

### Statistical analysis

Data analysis was performed using one-way analysis of variance (one-way ANOVA) for the comparison of pellet cartilage diameters and Mann-Whitney U for macroscopic and microscopic improvement evaluations by means of the SPSS software version 16 (IBM, USA). The P<0.05 was statistically considered significant. 

## Results

### Isolation and characterization of mesenchymal stem cells

**Fig.1:**
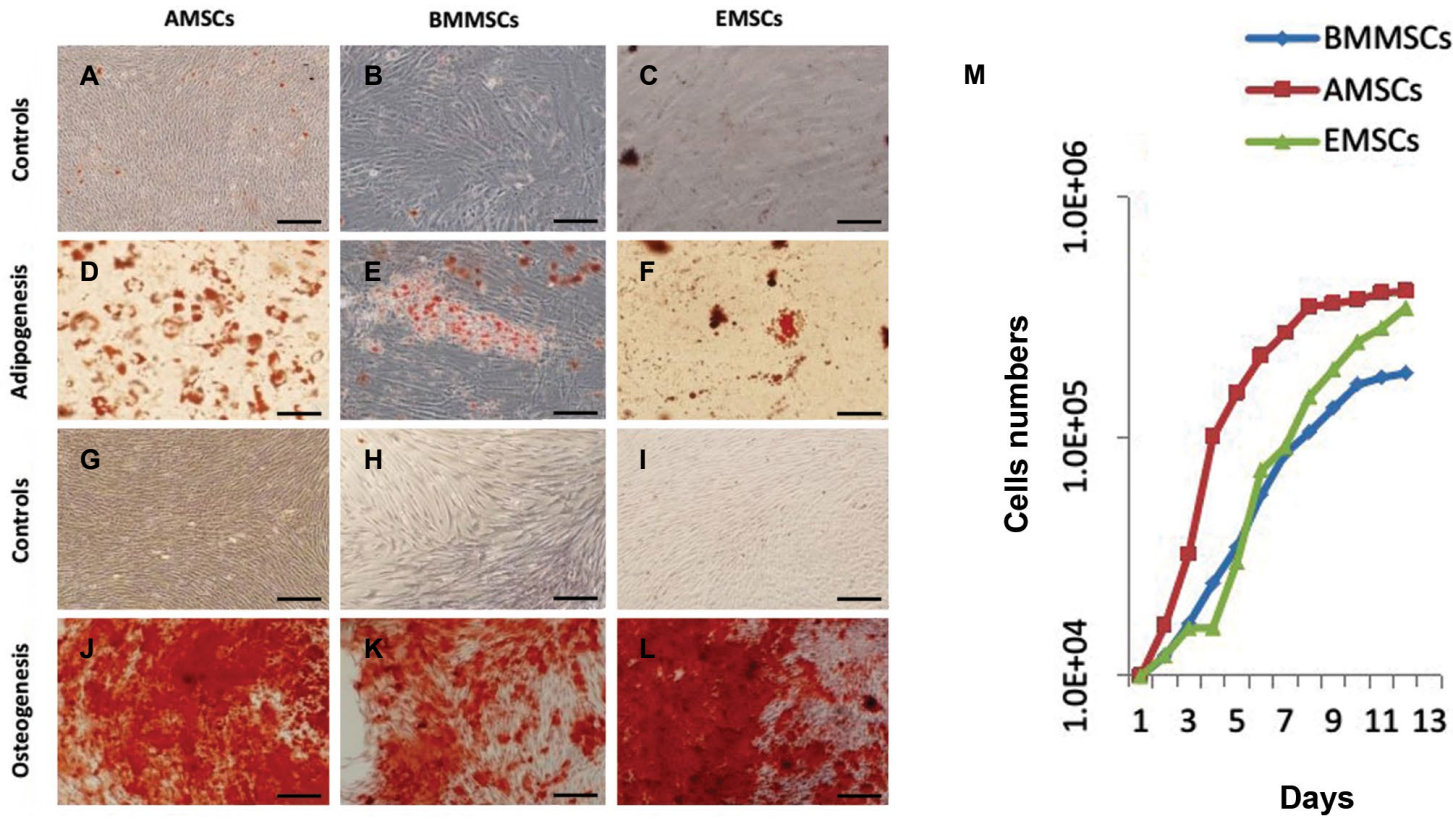
Evaluation of differentiation potential and growth rate of MSCs which were derived from adipose, the ears and bone marrow. **A-F. **Differentiation of extracted MSCs into adipocytes after oil-red staining (differentiation controls are shown on the top of the images, respectively) (scale bar: A: 200 µm, B: 100 µm, C, D: 50µm, E: 100 µm, F: 50 µm), **G-L.** Differentiation of extracted MSCs into osteoblast cells after alizarin red staining (differentiation controls are shown on the top of theimages, respectively) (scale bar: G-L: 200 µm), and **M.** The growth rate curve of the three MSCs which were derived from adipose, the ear, and bone marrow wereillustrated (cell counting using improved Neubauer Hemocytometer). MSC; Mesenchymal stem cells, AMSC; Adipose MSC, BMMSC; Bone marrow MSC, and EMSC; Ear MSC.

We isolated MSCs from the ear, adipose, bone marrow tissues, and expanded plastic adherent cells. The cells were spindle-shaped, fibroblast-like, and formed colonies. The differentiation of MSCs into adipocytes and osteoblast cell types was assessed by oil red and alizarin red (Fig .1A-L) staining, as well as qRT-PCR. The oil droplets existed in the culture plates indicated the adipogenesis (Fig .1D-F). As shown in Figure 1J-L, the mineral deposition occurred in all groups. 

Since the purpose of this study was to produce the cartilage tissue in a laboratory for transplantation, the cell growth rate was an important factor for saving time and cost. As shown in Figure 1M and PDT calculation, AMSCs had the highest rate of growth and proliferation, whereas the lower growth belonged to EMSCs and BMMSCs (34 hours versus 43 and 51 hours, respectively). 

**Fig.2:**
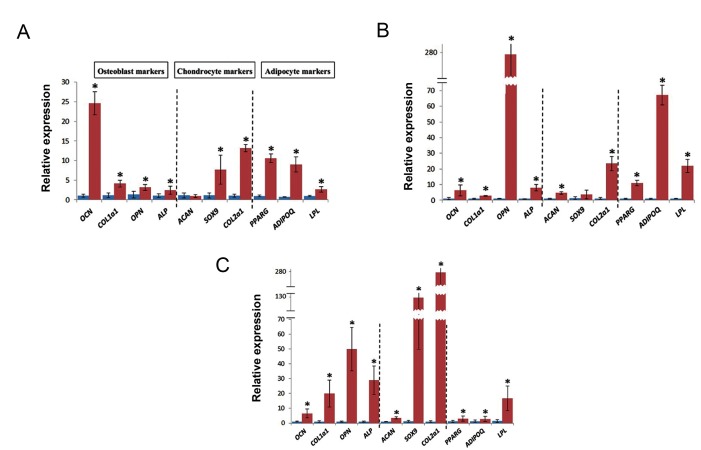
The expression profile of differential markers in differentiated versus undifferentiated cells. Expression analysis of **A.** AMSCs, **B.** BMMSCs, and **C.** EMSCs by real-time polymerase chain reaction. These results indicated the expression of differentiated genes compared to the undifferentiated cells. Adipocyte markers: *OCN, COL1a1, OPN,* and *ALP*. Chondrocyte markers: *ACAN, SOX9,* and *COL2a1*. Osteoblast markers: *PPARG, ADIPOQ,* and *LPL.* *; P<0.05 versus undifferentiated cells, error bar: means ± SD, n=5, AMSC; Adipose mesenchymal stem cell, BMMSC; Bone marrow MSC, and EMSC; Ear mesenchymal stem cell.

The expression profile of the gene markers of osteoblast, adipose, and cartilage tissues was investigated using RT-PCR. The results confirmed the expression of specific differentiation markers in these tissues (Fig .2). The expression of adipose differential markers by differentiated AMSCs showed a significant difference in *LPL, ADIPOQ,* and *PGAMA* gene expression. AMSCs also showed a significant difference in osteogenic gene expression namely OCN, *COL1a1, OPN,* and* ALP* after differentiation into the osteoblast. The differentiated AMSCs into chondrocytes only showed a significant difference in *SOX9* and *COL2a1* expression, but *ACAN* gene expression was not increased. Investigation of the gene expression in differentiated BMMSCs showed a significant difference in *OCN, COL1a1, OPN,* and *ALP* in differentiated osteoblast. *PPARG, ADIPOQ,* and *LPL* expressions also showed a significant difference in adipocytes which differentiate from BMMSCs. Differentiated chondrocytes from BMMSs showed a significant difference in ACAN and *COL2a1* gene expression, but not in *SOX9*. The analysis of the gene expression in differentiated EMSCs showed a significant difference in *PPARG, ADIPOQ,* and *LPL* in differentiated adipocytes. *OCN, COL1a1, OPN* and *ALP* gene expression in osteoblast originated from EMSCs showed a significant increase. *SOX9, COL2a1,* and to a lesser extend *ACAN* were significantly increased in chondrocytes which differentiate from EMSCs.

### Comparison of cartilage differentiation capacity among isolated mesenchymal stem cells 

**Fig.3:**
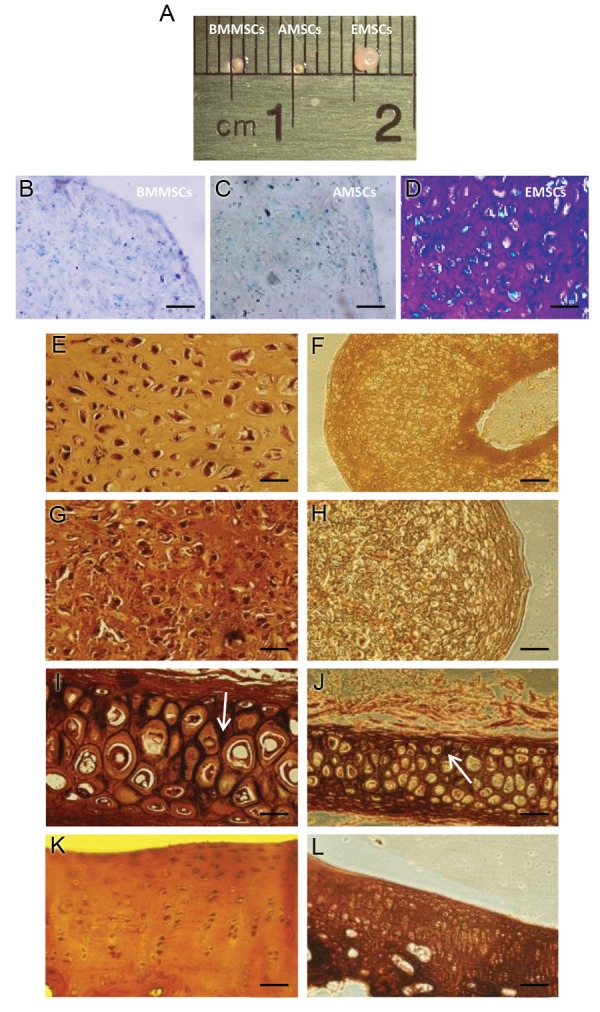
Size and microscopic structure of differentiated cartilage from three MSCs derived cells. **A.** Comparison the size of cartilage produced from BMMSCs, AMSCs, and EMSCs. EMSCs derived cartilage was significantly larger than other ones, **B-D.** Microscopic structure of cartilages following staining with toluidine blue. In EMSCs cartilage, more AGGRECAN production is obvious (scale bar: <bold>B, C:</bold> 100 µm, D: 50 µm),, and **E-L.** Verhoff staining of elastin strands in differentiated cartilage tissues from the EMSCs (E, F), BMMSCs (G, H), rabbit’s ear (I, J. as positive controls) where elastin fibers are well seen (white arrows) and the rabbit’s knee cartilage (K, L. as negative controls) in which elastin strands are not visible as well as E-H images. The arrows indicate the elastin deposited among the cells (scale bar: E, G, I, K: 50 µm, F: 200 µm, H: 100 µm, J, L: 500 µm). MSC; Mesenchymal stem cell, AMSC; Adipose MSC, BMMSC; Bone marrow MSC, and EMSC; Ear MSC.

All three cell lines underwent differentiation into chondrocyte lineage using the micro mass culture system. Figure 3A shows the size of produced cartilage from different MSCs. The average sizes of produced pellet cartilages were 0.683, 0.573, and 1.847 mm for BMMSCs, AMSCs, and EMSCs, respectively. The statistical analysis showed significant differences between groups in terms of the size. The microscopic structure of differentiated cartilage from three types of MSCs was investigated using toluidine blue staining, in which acidic proteoglycans (AGGRICANS) showed purple color (Fig .3B-D). Due to the small size of AMSCs pellet cartilage, it was excluded from the experimental groups. To analyze elastin fiber formation in produced pellet cartilages, Verhoff staining was performed (Fig .3E-L). No elastin strands were found in differentiated cartilages, and they were structurally similar to knee cartilage, which is a hyaline type.

### Macroscopic and microscopic assessments in different groups

**Fig.4:**
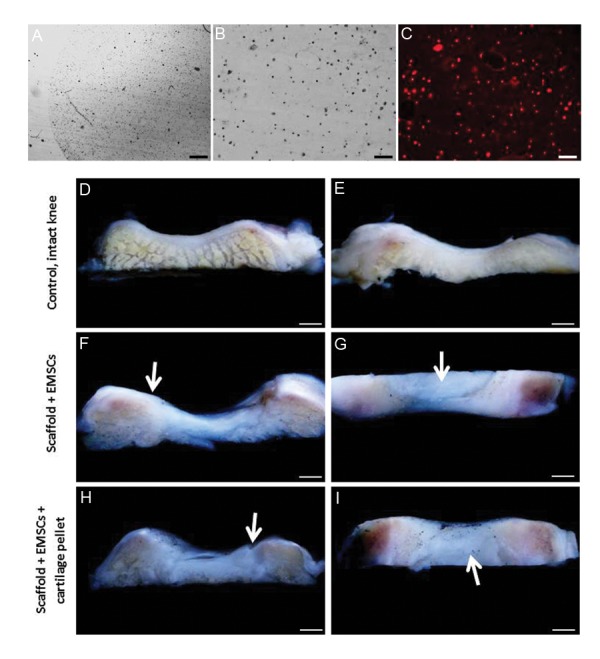
Distribution of MSCs in the scaffold and cross-sectional features of the trimmed knees. **A.** Microscopic view of cross-sections of the scaffold containing BMMSCs, in which a uniform distribution of cells is observed in scaffold (scale bar: 500 µm), **B, C.** The scaffold cross-section containing the stained cells with PKH26 (scale bar: 100 µm), **D, E.** Cross-sectional and upper facial features of the trimmed knees in the control (healthy knee), **F, G.** Knees receiving EMSCs/Scaffold, and **H,** **I.** Knees receiving EMSCs/Scaffold along with cartilage pellet. The arrows indicate the smoothness level of grafting surface (G and I) and the adhesion of the graftingtissue to adjacent tissues (F and H). (D-I: scale bars: 1 mm). MSC; Mesenchymal stem cell, BMMSC; Bone marrow MSC, and EMSC; Ear MSC.

For transplantation of cells and produced cartilages for thedefect sites in rabbits, collagen type I was used as a scaffold. Cross-sections of the MSC-seeded scaffold (collagen I) thatwere stained with PKH26 dye revealed a relatively uniform distribution of MSCs throughout a gel (Fig .4A-C). 

**Fig.5:**
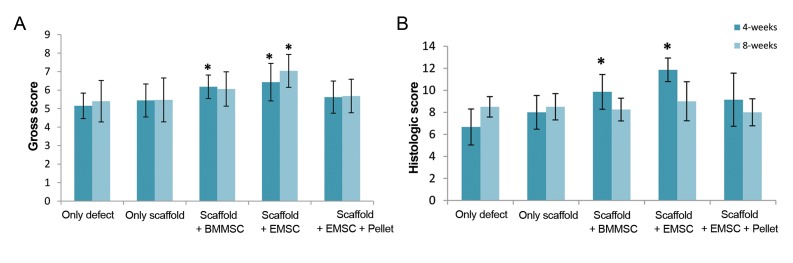
The results of the assessment forms and improvement score charts for the different groups in 4- and 8-week samples. **A.** The results of the assessment forms (n=18 each) showed a significant difference in scaffold+BMMSC or EMSC in 4-week groups and a significant difference in scaffold+EMSC in 8-week group and **B.** The histologic score of the different groups (n=6 each) showed a significant difference in scaffold+BMMSC or EMSC only in 4-week groups. A significant difference in the groups was shown with the only defect group. *; P<0.05 versus only defect group, BMMSC; Bone marrow mesenchymal stem cells, and EMSC; Ear MSC.

After transplantation, knees were removed and decalcified. The knee sections showed smoothness of grafting surfaceand the adhesion of the grafting tissue to adjacent tissues(Fig .4D-I). The macroscopic evaluation indicated that allstudied groups were improved compared to the control group4 weeks post-transplantation. However, only BMMSCs/ scaffold and EMSCs/scaffold showed a significant difference in terms of filling, color, and smoothness in the macroscopic scoring evaluation. The groups received MSCs/scaffold showed a significant difference in improvement of score compared to both the control and sham groups after 8-week (Fig .5). 

Based on microscopic scores, after 4 weeks post- implantation, the knees that received EMSCs/scaffold and BMMSCs/scaffold had higher scores than the other groups. This difference is significant when compared to the negative control (only defect). Also, in 8 weeks groups, there was a significant difference between the groups receiving EMSCs/ scaffold and the control. On the other hands, there was no significant difference among the 8 weeks post-implantation groups (Fig .5).

**Fig.6:**
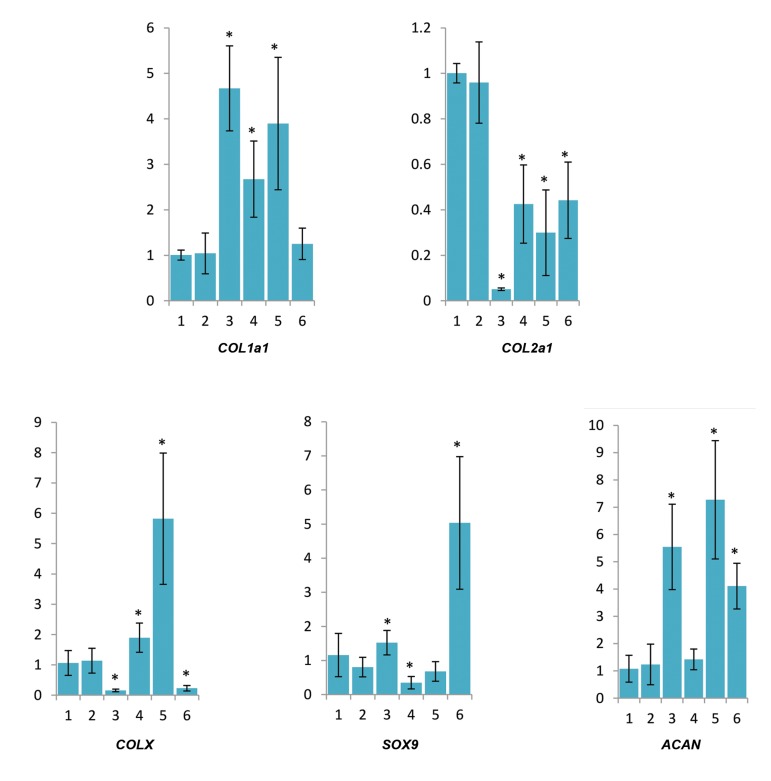
The evaluation of the expression of cartilage marker genes *in vivo* samples after 8 weeks in different groups. A significant difference in the groups was shown with the only defect group. *; P<0.05 versus only defect group, 1; Only defect, 2; Only scaffold, 3; Scaffold+BMMSCs, 4. Scaffold+EMSCs, 5; Sca+EMSC+pallet, and 6; Intact knee cartilage.

### Gene expression analysis

The expression profile of cartilage marker was analyzed 8 weeks post-implantation (Fig .6). The results indicated that there were no significant differences in all analyzed genes between the defect and scaffold alone samples. The expression level of *COL1a1* was up-regulated in all groups that received MSCs compared to intact cartilage, sham, and negative control. There was no significant difference between healthy knee, defect (negative control), and sham.

The expression level of *COL2a1* was significantly increased in the negative control and sham groups compared to the other groups. The groups receiving EMSC/scaffold and EMSC/scaffold with cartilage pellet did not show any significant difference in the expression of *COL2a1*. In contrast, the expression of *COL2a1* was substantially decreased in the BMMSC/scaffold. 

The gene expression of *COLX* was significantly down- regulated in the BMMSCs/scaffold and intact groups compared to both control and sham groups. However, the groups that received EMSCs/scaffold with and without cartilage pellet had a significant increase compared to the negative control.

The higher expression level of *SOX9* was detected in BMMSCs/scaffold in comparison with the negative control. The EMSCs/scaffold showed a significant reduction in the expression level of *SOX9*. With respect to the positive control, all groups showed a significant decrease in the expression of *SOX9*.

A significant increase in *ACAN* expression level was detected in BMMSCs/scaffold, EMSCs/Scaffold with cartilage pellet, and an intact knee compared to the other groups.

## Discussion

Nowadays, one of the major challenges in orthopedics is the treatment of AC injuries and mesenchymal stem cells are a promising cell source in regenerative medicine of the cartilage repair. Despite growing interest in the use of MSCs in preclinical research for AC regeneration, the translation into clinical settings is not satisfying. Although cell-based therapy is apparently simple in cartilage tissue due to the absence of an intrinsic capillary network and low density of one cell type, mechanical properties, and prestressed matrix make the cartilage more complicated (3). Previous systematic studies indicated the clinical benefit of MSCs therapies in most studies with no major adverse effects in the treatment or cell harvest (30, 31). However, several factors such as MSCs extraction technique, manipulation, and the release of the cells, as well as the optimization of cellular dose are the challenges ahead. On the other hand, the heterogeneity and lack of defined standards in studies caused using various strategies. Therefore, specific studies to find the best cell source and how to manage the manipulation of the cells, as well as the release techniques and the indication of pathology are necessary in order to achieve an effective treatment.

Our results showed that all three types of the isolated MSCs were differentiated into bone, adipose, and cartilage that confirmed the mesenchymal phenotype of the extracted cells. The cell growth rate is of great importance in tissue engineering in order to shorten the process time and decrease the expenses. The comparative curve of the cell growth and its doubling time indicated that AMSCs grew faster than BMMSCs and EMSCs. Therefore, these cells could be suitable candidates for cartilage tissue engineering. In addition to the growth rate, differentiation potential into chondrocytes and chondrogenic gene expressions are the other determinative factors. EMSCs and BMMSCs produce bigger pellets in comparison with AMSCs, which are in agreement with the previous studies. The results of qPCR showed that the expression of ACAN in AMSC and SOX9 in BMMSC did not show any significant differences. Significant differences were found in the expression of SOX9, ACAN, and COL2a1 in EMSCs, confirming the higher capability of EMSCs for chondrogenic differentiation. Thus, AMSCs, in spite of their simple harvesting and rapid growth, could not be a proper cell source in this study according to the results of the differentiation of these cells into pellets cartilage. The ability of AMSCs for differentiation into chondrocyte is supposedly improved by the alteration of the induction medium composition (32). Interestingly, depending on the extraction and differentiation methods, EMSCs seem to have a better potential for the differentiation into cartilage.

The differentiation of MSCs into the cells of tissues that they are originated from is one of the main concerns in tissue engineering. Interestingly, Verhoeff-van Gieson staining confirmed the absence of elastin strands in the differentiated cartilage obtained from EMSCs and BMMSCs. Elastic fibers are abundant in the elastic cartilage of the ear and invisible in hyaline cartilage. It appears that ear-derived MSCs can effectively differentiate into hyaline cartilage, as Mizuno et al. reported the potential of the ear-derived cartilage progenitor cells in the reconstruction of joint hyaline cartilage (33). 

In cell-based cartilage therapy, some issues such as the safety of MSCs and viability of the cells before transplantation should be considered. Although, techniques are being developed throughout the world and the safety of MSCs has been proven in ongoing clinical trials, but, basic studies seeking suitable cell source and approving a valid methodology and regenerative intervention can reduce many concerns. We sought to address the chondrogenic potential of the isolated cells in cartilage defects. Macroscopic evaluation of the defect site indicated that all cell/scaffold groups led to cartilage regeneration, though the EMSCs/scaffold improved the lesion more quickly 4 weeks post-transplantation. Eight weeks after transplantation, there was no significant improvement which might be related to the inherent regeneration ability. Indeed, cartilage in rabbits, unlike human, has an inherent repair ability that affects many analyses (34). The histological analysis revealed a higher degree of defect regeneration in all cell/scaffold groups than the only defect group 4 weeks post-transplantation. These results confirmed the macroscopic results, while in microscopic scoring the 8-week groups, there was no significant difference between the groups and the control group. Of note, the addition of a pellet to cell/scaffold not only did not improve the outcomes but also had a negative effect on EMSCs/scaffold. Previse experiments have shown the positive effects of cartilage fragment in cartilage regeneration (35). Our findings are not in agreement with this notion. This difference could be due to different materials (cartilage fragment versus MSCs palette) used in these studies. Based on these results, it is concluded that the use of the rabbit’s knee is a convenient model for the short-term (first four weeks) *in vivo* studies, and after that, the inherent regeneration system would repair the cartilage defect.

The *SOX9* gene, a master transcription factor, is the main enhancer of specific cartilage genes such as *COL2a1, COLIXa1, COLXIa2, ACAN, *cartilage binding protein, and COMP (36). Downregulation of *SOX9* in our results may be related to the expression of inflammatory factors such as interleukin-1ß and TNF-α in the defect site, as the expression of these factors has a negative effect on the expression of the *SOX9* (37) and *COL2a1*. Aggrican is the main proteoglycan of the cartilage extracellular matrix which could indicate a severe chondrogenesis activity (38) in scaffold/BMMSC and scaffold/EMSC/ pellet groups. Although *SOX9* is the upstream regulator of ACAN, there are additional pathways and transcription factors that regulate *ACAN* expression (39). Collagen X is a marker of cartilage cells present in hypertrophic stage and leads to the bone formation (40), which could indicate hypertrophy of cartilage cell in scaffold/EMSC and scaffold/EMSC/pellet groups. However, here, we used collagen type I as a scaffold that exists in bone tissue, skin, and tendon. The expression of collagen type I gene and COL2a1 in cartilage-transplanted tissues could represent the fibrosis of these structures and cross-talk of the ECM and transplanted cells. Taken together, in our *in vivo* gene expression analyses, we should consider the expression time and complex signaling crosstalk in chondrogenesis.

## Conclusion

Despite the improvements in regenerative medicine, the application of cell-based cartilage therapy in clinic remains complex. Here, we compared chondrogenesis potential of bone marrow-, adipose-, and the ear-derived MSCs *in vitro* and *in vivo*, and showed the different characteristics and regeneration capacity of these cell sources. AMSCs have the highest proliferation rate, but lowest differentiation potential to cartilage compared with EMSCs and BMMSCs. Furthermore, EMSCs showed the highest chondrogenic potential as shown in the gene expression and histologic assessments. These results confirmed the importance of cell source selection with respect to *in vivo* cartilage regeneration. In line with this, EMSCs would be an appropriate option for promoting cartilage reconstruction. Moreover, since the ear tissue could be easily harvested from a cadaver, it would be a valuable substitute for MSCs from bone marrow tissue. Overall, these findings could be used to improve the strategies in cell-based cartilage therapy. 

## Supplementary PDF


