# Supplementary material for: In Vitro and In Vivo Comparison of Different Types of Rabbit Mesenchymal Stem Cells for Cartilage Repair
Source: Cell J. 2019 Feb 25;21(2):150–60. doi: 10.22074/cellj.2019.6149. (PMC6397606; doi:10.22074/cellj.2019.6149.)
Supplement: Supplementary file 1 [file Cell-J-21-150-s01.pdf]

CELL JOURNAL (Yakhteh)

Supplementary Information for

In Vitro and *In Vivo* Comparison of Different Types of Rabbit Mesenchymal Stem Cells for Cartilage Repair

Mohammad Ali Khalilifar, M.Sc.[1, 2], Mohamadreza Baghaban Eslaminejad, Ph.D.[1]*, Mohammad Ghasemzadeh, Ph.D.[3], Samaneh Hosseini, Ph.D.[1], Hossein Baharvand, Ph.D.[1, 2]

1. Department of Stem Cells and Developmental Biology, Cell Science Research Center, Royan Institute for Stem Cell Biology and Technology, ACECR, Tehran, Iran
2. Department of Developmental Biology, University of Science and Culture, Tehran, Iran
3. Infertility and Reproductive Health Research Center, Health Research Institute, Babol University of Medical Sciences, Babol, Iran

Corresponding Address: P.O.Box: 16635-148, Department of Stem Cells and Developmental Biology, Cell Science Research Center, Royan Institute for Stem Cell Biology and Technology, ACECR, Tehran, Iran
Email: eslami@royaninstitute.org

Table S1: Primer sequences used for real-time polymerase chain reaction

Code	Molecule	Primers (5´-3´)	Product size (bp)	Annealing temp. (˚C)
Rb13	*COL2a1*	F: AATCCCAGAACCCATCAGG<br>R: CCAGTAGTCACCGGTCTTCC	325	56
Rb 12	*SOX9*	F: AAGATGACCGACGAGCAG<br>R: GGCTTGTTCTTGCTGGAG	284	56
Rb14	*ACAN*	F: GGAGGTCGTGGTGAAAGGTG<br>R: CTCACCCTCCATCTCCTCTG	255	61
Rb11	*GAPDH*	F: GACCACCATCCATTCCTACA<br>R: GCAAGTCAGGTCCACAACAG	276	60
rRb 17	*ALP*	F: ACTTTGTCTGGAACCGCACT<br>R: GTGGTCAATCCTGCCTCCT	215	58
rRb 19	*OPN*	F: GGCTAAACCCTGACCCATCT<br>R: GTGGTCATCGTCCTCATCCT	161	59
rRb 18	*OCN*	F: CTCAGCCTTCGTGTCCAA<br>R: CTCGCACACCTCCCTCTTG	126	57
Rb 4	*COL1a1*	F: GGT GCT GCT GGT AAA GAA GG<br>R: GTC TAC CCA AAG CAC CAG	142	60
Rb 7	*COLX*	F: GCC GAC CAA GGA AAT GAC<br>R: GTA ACA GCC AGT CCA CCA C	267	60
rRb 20	*LPL*	F: TTCAACCACAGCAGCAAGAC<br>R: TAACAGCCAGTCCACCACAA	141	57
rRb 21	*ADIPOQ*	F: CGGTGAGAAGGGTGAAAAAG<br>R: GCTGAGCGGTAGACATAG	156	57
Rb 10	*PPARG*	F: CAG GAG CAG AGC AAA GAA GT<br>R: GTC CGT CTC CGT CTT CTT TA	570	56

Table S2: Scoring system used for macroscopic evaluation of repaired defects in cartilage

Criterion	Macroscopic characteristic	Points
Filling	Distinct underneath surrounding cartilage	1
	Up to surrounding cartilage ,central depression	2
	Flush with surrounding cartilage	3
Color	Brown or yellow	1
	White	2
	Same as surrounding cartilage	3
Surface	Rough	1
	Smooth	2
Overall score		8

Table S3: Histological scoring system for evaluation of repaired defects in cartilage

**Cell morphology**		**Thickness of cartilage (%)[b]**	
Hyaline cartilage	4	121–150	1
Mostly hyaline cartilage	3	81 - 120	2
Mostly fibro cartilage	2	51–80	1
Mostly non-cartilage	1	0–50	0
Non-cartilage only	0	Integration with adjacent cartilage	
Matrix-staining (meta chromasia)		Both edges integrated	2
Normal	3	One edge integrated	1
Slightly reduced	2	Neither edge integrated	0
Markedly reduced	1	Total maximum	14
No metachromatic staining	0		
Surface regularity[a]			
Smooth	3		
Moderate	2		
Irregular	1		
Severely irregular	0		

[a]; Total smooth area of the reparative cartilage compared with the entire area of the cartilage defect and [b]; Average thickness of the reparative cartilage compared with that of the surrounding area.

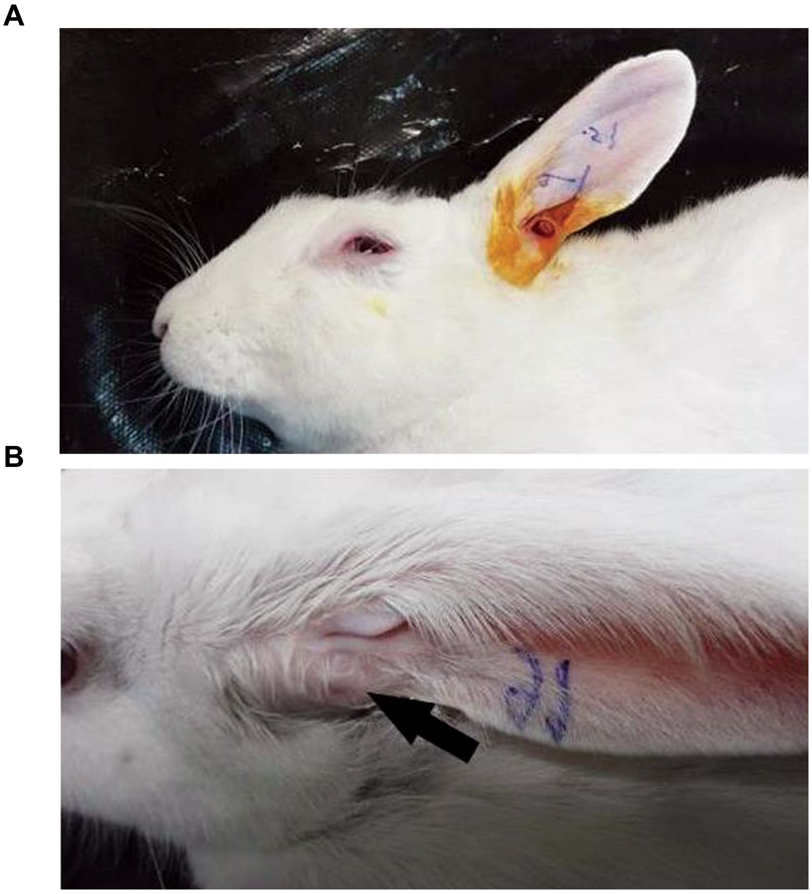

Fig.S1: Rabbit ear punching for the isolation of EMSCs and healing. **A.** The wound area was disinfected with oxytetracycline spray after punching and **B.** The ears of rabbits healed after 8 weeks because of the high intrinsic regeneration potential (black arrow).

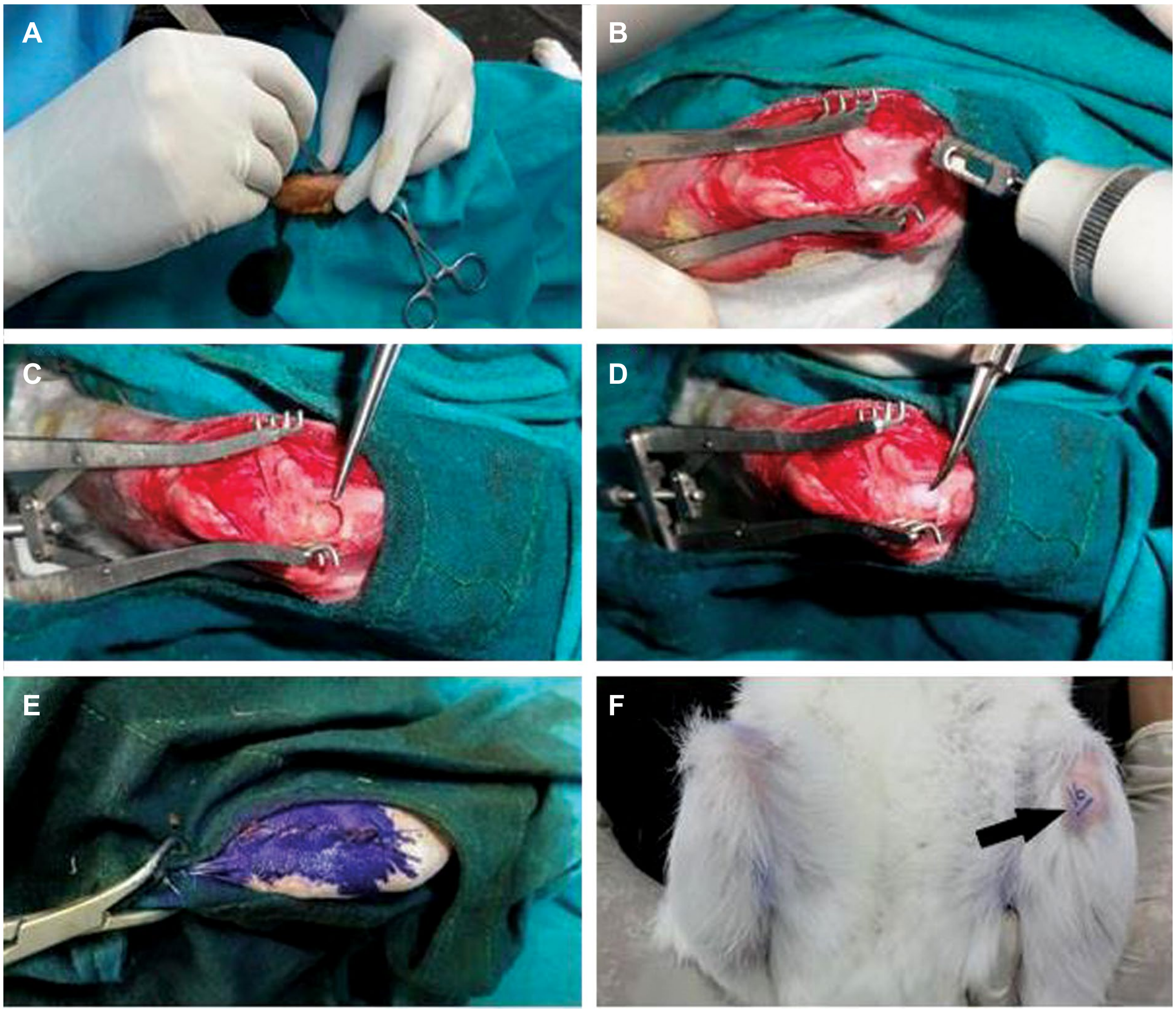

Fig.S2: Different stages of knee surgery and transplantation. **A.** Medial parapatellar incision, **B.** Retraction of patella, articular capsule and skin and make defect with dental trephine, **C.** The defect, **D.** Transplantation of the scaffold and the cells, **E.** Sutures the capsule and skin, layer by layer, and **F.** Wound healing and repair.
